# Neurological soft signs in persons with amnestic mild cognitive impairment and the relationships to neuropsychological functions

**DOI:** 10.1186/1744-9081-8-29

**Published:** 2012-06-07

**Authors:** Hui-jie Li, Peng-yun Wang, Yang Jiang, Raymond C K Chan, Hua-li Wang, Juan Li

**Affiliations:** 1 Center on Aging Psychology, Key Laboratory of Mental Health, Institute of Psychology, Chinese Academy of Sciences, Beijing, China; 2 Graduate School, Chinese Academy of Sciences, Beijing, China; 3 Department of Behavioral Science, College of Medicine, University of Kentucky, Kentucky, USA; 4 Neuropsychology and Applied Cognitive Neuroscience Laboratory, Key Laboratory of Mental Health, Institute of Psychology, Chinese Academy of Sciences, Beijing, China; 5 Department of Geriatric Psychiatry, Institute of Mental Health, Peking University, Beijing, China

**Keywords:** Mild cognitive impairment, Neurological soft signs, Neuropsychological tests

## Abstract

**Background:**

Neurological abnormalities have been reported in people with amnestic mild cognitive impairment (aMCI). The current study aimed to examine the prevalence of neurological soft signs (NSS) in this clinical group and to examine the relationship of NSS to other neuropsychological performances.

**Methods:**

Twenty-nine people with aMCI and 28 cognitively healthy elderly people were recruited for the present study. The NSS subscales (motor coordination, sensory integration, and disinhibition) of the Cambridge Neurological Inventory and a set of neuropsychological tests were administered to all the participants.

**Results:**

People with aMCI exhibited significantly more motor coordination signs, disinhibition signs, and total NSS than normal controls. Correlation analysis showed that the motor coordination subscale score and total score of NSS were significantly inversely correlated with the combined Z-score of neuropsychological tests in aMCI group.

**Conclusions:**

These preliminary findings suggested that people with aMCI demonstrated a higher prevalence of NSS compared to healthy elderly people. Moreover, NSS was found to be inversely correlated with the neuropsychological performances in persons with aMCI. When taken together, these findings suggested that NSS may play a potential important role and serve as a tool to assist in the early detection of aMCI.

## Introduction

Alzheimer’s disease (AD) is the most commonly acquired neurodegenerative disease in elderly people [1]. Mild cognitive impairment (MCI) is considered as a transitional state of normal aging and AD [2], and was also thought to be the earliest clinical manifestation of common age-related neurological abnormalities, including AD [3].

Neurological abnormalities have traditionally been divided into “hard signs” and “soft signs” [4]. “Hard signs” usually indicate focal neurological deficits localized within specific brain regions, whereas “soft signs” are conventionally defined as subtle signs without an identifiable or localized brain region [4]. However, most recent studies using brain imaging technologies suggested that neurological soft signs (NSS) might be associated with specific brain regions or even brain connections [5-8]. For example, researchers found that higher rates of motor coordination and sensory integration signs were associated with a reduction of grey matter volume of subcortical structures, including putamen, globus pallidus and thalamus in both patients with first-onset schizophrenia and healthy volunteers [5,6]. Chan et al. (2006) also showed that brain areas such as bilateral sensorimotor, supplementary motor area, left parietal, and right cerebellum were activated during the fist-edge-palm task [7]. Furthermore, the fist-edge-palm task as a soft sign for motor coordinaiton has been shown to be linked to connectivity between the right inferior and middle prefrontal cortices in healthy control subjects [8].

Patients with AD have also been found to demonstrate significantly higher prevalence of sensory integration and motor coordination signs than healthy controls [9]. Other studies also found NSS abnormalities in patients with AD [10,11]. Kumamoto et al. (2000) found elderly people that were cognitively impaired but not demented, had exhibited higher frequency of neurological signs than healthy controls, but lower rate of signs than patients with dementia [12]. Gualtieri et al. (2005) also found that individuals with MCI showed poorer performance in a finger tapping task in which participants were required to tap on the mouse button [13]. A previous study implied that NSS might be a predictor for progression to clinical AD [14]. Yet so far there is no empirical study to systematically explore the NSS in individuals with aMCI. In the present study, we hypothesized that individuals with aMCI would demonstrate more NSS than normal elderly individuals, and that NSS might be taken as an easy-identified neurological marker for early detection of AD.

Neuropsychological performance is considered to be one of the most sensitive and specific markers of prodromal AD [15]. Previous studies indicated that cognitive decline in patients with MCI was associated with widespread structural brain damage [16]. As we know, the executive function aging is the main cause of the cognitive decline in older adults. The so called “frontal hypothesis of cognitive aging” suggests that the prefrontal cortex deteriorated earlier and disproportionately compared to other cortices [17]. In addition, the pathological aging causes declines in volume and microstructural pathology in prefrontal cortex, medial temporal regions, and other regions in grey and white matter density [18-21]. Thus, the motor coordination, sensory integration, and disinhibition subscales of the Cambridge Neurological Inventory (CNI) have been devised to investigate the putative regions of prefrontal lobe, parital lobe, and frontal lobe, respectivelly [22,23]. Studies using sturcutral equaiton modelling showed that there were modest and moderate associations among NSS, executive function, verbal memory, and visual memory in both patients with schizophrenia and healthy controls. Moreover, NSS has been associated with poorer performances in executive function and memory functions in both groups [24]. Recently, Chan et al. (2011) also observed a similar pattern for relationship in healthy older adults [25]. When taken together, these findings suggest that neurolgoical soft signs are capable to capture the similar information measured by conventional neurocognitive tests.

To date, it is still largely unknown about the prevalence rate of NSS in people with MCI and how these signs are related to conventional neuropsychological performances in this clinical group. The purpose of the current study was to explore the prevalence of NSS in aMCI and to examine the relationships of NSS to neuropsychological performances in this clinical group. Given the aforementioned studies, it was hypothesized that aMCI was associated with a higher prevalence of NSS as compared to healthy older adults. Moreover, NSS was expected to show negative association with neuropsychological performances in this clinical group.

## Methods

### Subjects

Patients with aMCI in this study were recruited from the communities around the Institute of Psychology, Chinese Academy of Sciences and memory clinic of Institute of Mental Health, Peking University. One hundred and sixty-seven participants aged between 60–87 years were screened from the residential communities. Among them, 18 were screened as patients with aMCI. Moreover, 11 individuals with aMCI were referred by the psychiatrists of the memory clinic. The diagnoses of aMCI were made according to published criteria [2,26]. Participants were interviewed to determine whether they had memory complaint, normal general cognitive function (measured by Mini-Mental State Examination, MMSE) [27], and normal activities of daily living [28]. Objective memory impairment was verified with paired-association learning and portrait characteristics recall tests, which were two subtests of the standardized Clinical Memory Scale [29,30]. For the cut-off scores of these two memory tests, we used a more liberal criterion of 1SD below the norms, as previous studies suggested that the traditional 1.5 SD cut-off would reduce the possibility of detecting early stage memory impairment [31]. An expert team, consisting of psychiatrists and neuropsychologists, made consensus diagnoses on the basis of all available clinical and neuropsychological results.

Healthy controls were also recruited from the same geographic region where people with aMCI were recruited. They were matched for age, gender, and education level with aMCI participants. They were also defined by having normal scores on the paired-association learning and portraits characteristics recall [29,30], activities of daily living [28], MMSE [27], and had no complaints of memory problems.

Participants were excluded for both groups if they: (1) had a history of head trauma; (2) had either a disease of the central nervous system or a psychotic disorder; (3) abused alcohol or other substance; or (4) were diagnosed with any form of dementia.

The present study was approved by the ethics committees of the Institute of Psychology, Chinese Academy of Sciences. Written informed consent was obtained from all participants.

### Neurological soft signs

The motor coordination, sensory integration, and disinhibition subscales of the Cambridge Neurological Inventory (CNI) was used to assess NSS [22]. The CNI is one of the most commonly used tools to explore the NSS and it has been validated among the Chinese population [32]. The motor coordination subscale includes items assessing rapid motor movements such as finger tapping, finger-thumb opposition, diadockinesia, fist-edge-palm test, and oseretsky test. The sensory integration subscale consists of items evaluating tactile sensation such as extinction test, finger-agnosia, stereognosis, graphesthesia, and left-right orientation. The disinhibition subscale includes items for withholding or inhibiting associated movements. These items include saccade blink and saccade head, wink, go/no-go test, and mirror movements of finger-thumb opposition (left and right hands) and diadocokinesia (left and right hands) [22,23]. The CNI has full instruction for training guidelines, and it also has been shown with good construct and external validity, and inter-rater reliability [23].

The CNI was administered in a standardized manner according to a fixed order. In the original scale, scoring was made according to standardized anchor points to indicate “normal” response (scored as 0), “equivocal response” (0.5), “abnormal” response (1) or “grossly abnormal” response (2). In the present study, item scores were dichotomized into either “absent” (covering normal or equivocal) or “present” (covering abnormal or grossly abnormal) [24].

### Neuropsychological tests

Participants also received a battery of neuropsychological tests capturing their capabilities in executive function, processing speed, abstract reasoning ability, and memory.

Fluency and Trail-making test (TMT) B were used to assess the executive function. The fluency test includes verbal fluency [33] and writing fluency testing. For verbal fluency, participants were told to speak as many animal names or food names as they could in one minute, respectively. The mean score was considered to be the performance of verbal fluency. For writing fluency, participants were told to write as many Chinese characters as they could for radicals of “扌” and “亻” in a minute, respectively, and the average score was considered to be the performance of writing fluency. For TMT B, 25 circles were printed in black on paper, including the first 13 Arabic numerals and the first 12 Chinese numbers. Participants were asked to connect Arabic numbers and Chinese numbers alternately (for example, 1-一, 2-二, 3-三, etc.), the last link is from the 十二 (12 in Chinese number) to the 13 [34].

Digit span subtest of Wechsler Memory Scale-Revised Chinese version (WMS-RC) was used to assess working memory, and both forward and backward tests were included [35]. Processing speed was evaluated by the digit symbol subtest of Wechsler Adult Intelligence Scale-Chinese version (WAIS-C) [36] and TMT A [34]. Similarity subtest of WAIS-C [36] was considered to investigate the verbal abstract reasoning ability, and it was considered to reflect the function of the frontal lobe [37]. Episodic memory was measured by the logic memory, which was selected from WMS-RC [35]. Participants were required to listen to two short stories and then to recall them immediately; the memory performance was evaluated with the average score of the two stories.

### Statistical analysis

Statistical analyses were carried out with the Statistical Package for Social Sciences (SPSS) version 13.0. First, Chi-square test was performed to compare the prevalence rate of NSS in patients with aMCI and healthy elderly people. Second, since years of education was significant between two groups, it was controlled as a covariate in analyses. A MANCOVA was used to analyze the group differences in NSS with Bonferroni correction. The effect sizes of the group comparisons were calculated in terms of Cohen’s d [38]. Finally, Pearson correlation analysis was used to test the relationships between NSS and neuropsychological performance in patients with aMCI.

## Results

### Demographics

Twenty-nine patients with aMCI (11 males) and 28 normal control participants (15 males) were included in the present study. The Chi square test indicated that there was no significant group differences in gender ratio (*χ*^2^ = 1.41, *p* = .29). As demonstrated in Table 1, there were no significant group differences in age and activities of daily living. Patients with aMCI received significant lower years of education. They also showed significant lower performance in MMSE, the paired-association learning and portrait characteristics recall tests. We also examined the medical histories in the two groups. For aMCI group, 21 out of 29 older adults reported medical histories, among them 14 suffered from hypertension and/or diabetes mellitus and/or coronary heart disease, 3 had cataract or other strabismus, 2 had cavity infarction, and 2 had benign prostate hypertrophy or mammary gland hyperplasia. Nineteen of the 28 healthy older adults reported medial histories. Specially, 14 had hypertension and/or diabetes mellitus and/or coronary heart disease, 2 had ocular fundus disease or astigmatism, 2 had cervical vertebral disease or stomach disease, and 1 had sleep disorder. The Chi square test indicated that there was no significant group differences in the ratio of suffering from chronic diseases (*χ*^2^ = .14, *p* = .78). For the neurocognitive tests, significant group differences were found in verbal fluency, digit symbol, TMT B, logic memory, and similarity, while no significant differences were found in writing fluency, TMT A, forward and backward of digit span.

**Table 1:** Demographic variables and neuropsychological tests for aMCI patients and normal older adults

	**aMCI**			**Normal Older Adults**			** *t* **	** *p* **
	** *N* **	** *M* **	** *SD* **	** *N* **	** *M* **	** *SD* **		
Age (years)	29	73.76	6.42	28	71.25	6.43	1.47	.15
Education (years)	29	10.97	4.82	28	13.89	3.12	−2.71	.009
Activities of daily living	29	14.93	1.36	28	14.54	1.37	1.09	.28
MMSE	29	26.07	2.33	28	27.61	1.95	−2.70	.009
Paired-association learning	29	4.45	3.06	28	11.14	3.29	−7.95	<.001
Portraits characteristics recall	29	4.00	3.76	28	9.39	5.09	−4.56	<.001
**Neuropsychological tests**								
Verbal fluency	29	18.81	4.35	28	22.64	4.26	−3.36	.001
Writing fluency	27	4.61	1.95	26	5.23	1.93	−1.16	.25
Digit symbol	27	30.30	10.50	27	35.81	7.53	−2.22	.031
TMT A (seconds)	29	51.07	23.59	24	44.21	16.20	1.21	.23
TMT B (seconds)	28	113.11	75.05	24	78.58	35.94	2.06	.045
Digit span-forward	26	7.31	1.64	27	7.85	1.32	−1.33	.19
Digit span-backward	26	4.15	1.32	27	4.59	1.72	−1.04	.30
Logic memory	27	5.06	2.51	27	8.44	2.89	−4.60	<.001
Similarity	29	15.41	4.20	28	18.36	3.28	−2.94	.005

### Comparison of NSS between older adults with aMCI and normal controls

The prevalence rate of “present” NSS in aMCI and normal controls are shown in Table 2. The Chi-square test showed that people with aMCI exhibited higher prevalence rate of NSS in most of the items, especially in fist-edge-palm in left hand, oseretsky test in motor coordination subscale, and go/no-go in disinhibition subscale.

**Table 2:** Prevalence rate of individual items of neurological signs in persons with aMCI (n = 29) and normal control (n = 28)

**NSS items**	**aMCI**	**Normal control**	** *χ* **^2^	** *p* **
**Motor coordination**				
Left Finger-thumb tapping	20.7%	0%	6.48	**.023**
Right Finger-thumb tapping	13.8%	3.6%	1.86	.35
Left Finger-thumb opposition	27.6%	21.4%	.29	.76
Right Finger-thumb opposition	17.2%	10.7%	.50	.71
Left Diadocokinesia	17.2%	10.7%	.50	.71
Right Diadocokinesia	13.8%	7.1%	.67	.67
Left Fist-edge-palm	51.7%	10.7%	11.01	**.001**
Right Fist-edge-palm	27.6%	7.1%	4.12	.079
Oseretsky test	58.6%	28.6%	5.22	**.033**
**Sensory integration**				
Extinction	10.3%	3.6%	1.00	.61
Left Finger Agnosia	69.0%	42.9%	3.94	.064
Right Finger Agnosia	34.5%	39.3%	.14	.79
Left Stereognosia	0%	0%	NA	NA
Right Stereognosia	0%	0%	NA	NA
Left Graphesthesia	13.8%	25.0%	1.15	.33
Right Graphesthesia	6.9%	10.7%	.26	.67
Left-Right Orientation	13.8%	14.3%	.003	.99
**Disinhibition**				
Left Mirror Movement of Finger Opposition	6.9%	0%	2.00	.49
Right Mirror Movement of Finger Oppostion	10.3%	3.6%	1.00	.61
Left Mirror Movement of Diadocokinesia	3.4%	3.6%	.001	.99
Right Mirror Movement of Diadocokinesia	24.1%	7.1%	3.10	.14
Saccade Blink	17.2%	7.1%	1.35	.42
Saccade Head	27.6%	10.7%	2.60	.18
Wink	10.3%	21.4%	1.32	.30
Go No-Go Stimulus	44.8%	10.7%	8.21	**.007**

*p*-values: two sided, Fisher’s Exact Test.

**Figure 1:**
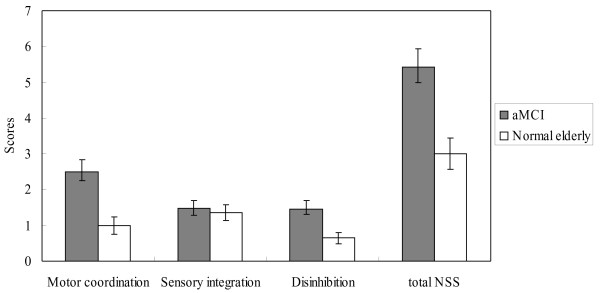
Comparisons of neurological soft signs in persons with aMCI and normal elderly. Error bars represent 95% confidence intervals.

The MANCOVA results indicated that patients with aMCI demonstrated significantly more dysfunctions in motor coordination (*F* (1, 54) = 6.95, *p* = .011), disinhibition (*F* (1, 54) = 6.78, *p* = .012), and the total NSS (*F* (1, 54) = 8.25, *p* = .006). For the sensory integration, there was no significant differences between the two groups (*F* (1, 54) = .06, *p* = .82). Figure 1 shows the NSS profiles of patients with aMCI and healthy controls. The effect sizes for motor coordination (Cohen’s d = 0.93) and total NSS score (Cohen’s d = 0.96) reached large, while the effect sizes were moderate for disinhibition (Cohen’s d = 0.76) and negligible for the sensory integration (Cohen’s d = 0.12).

### Correlations between NSS and neuropsychological tests in patients with aMCI

The neuropsychological performance was indexed by the combined Z-score of the verbal fluency and writing fluency, digit symbol, forward and backward of digit span, TMT A and B, similarities, and logic memory.

Pearson correlation analysis showed that the total NSS score and motor coordination subscale were significantly negatively correlated with combined Z-score of neuropsychological tests (*r* = −.53, *p* < .01; *r* = −.42, *p* < .05), the sensory integration and disinhibition subscales were not found to be significantly correlated with combined Z-score of neuropsychological tests (*r* = −.19, *p* > .05; *r* = −.33, *p* > .05).

## Discussion

Our results revealed that patients with aMCI exhibited a higher prevalence rate of neurological abnormalities than normal control participants. The preliminary results also suggested NSS and neuropsychological tests might reflect somewhat similar information for the brain functioning. The present study indicated that NSS may play an important role and serve as a tool to assist in the early detection of aMCI.

To our knowledge, this is the first study to investigate the NSS in older adults with aMCI. Our results demonstrated that aMCI individuals displayed significantly more neurological abnormalities than normal controls on motor coordination, disinhibition, and the total NSS. The effect sizes of group comparisons were large for motor coordination and total score and moderate for disinhibition. The findings were similar to previous studies focused on normal elderly people and people with pathological aging disease. With the same scale, Chan et al. (2011) found that NSS was common among elderly people, and the prevalence rate of soft signs increased with advancing age [25]. In a neurological examination including few soft signs (etc., saccadic eye movement), the older adults who were with cognitively impaired but without dementia were found to produce a higher prevalence of signs than the normal controls [12]. Patients with AD and other several forms of dementia were also found to have higher prevalence of NSS than those without dementia [9].

The current study indicated that older adults with aMCI showed more motor dysfunctions than cognitively normal older people. Previous findings indicated that people with MCI and mild AD demonstrated dysfunctions in equilibrium and limb coordination [39]. Other studies confirmed that people with aMCI performed worse on tasks involving fine and complex motor functions than normal older adults [40,41]. Lam et al. (2005) also found motor coordination signs were very sensitive in discriminating patients with or without dementia [9]. Signs such as primitive reflexes and mirror movements were classified as disinhibition, which included the signs of spurious movements in a time and place where it was not expected to occur [22]. In the present study, patients with aMCI demonstrated significantly more signs than normal controls in the disinhibition subscale. Similar findings were also reported in previous studies. Franssen et al. (1991) found participants in an early stage of AD showed higher mean score of deep tendon reflexes than normal elderly people, while patients with a later stage of AD demonstrated significantly increased prevalence of sucking reflexes compared with normal older adults and patients with the early stage of AD [10]. Furthermore, more primitive reflexes were found in the more terminal stages of AD [11]. Previous studies also found that MCI patients showed inhibition impairments in some neuropsychological tasks, such as go/no-go task [42], Stroop task [43,44], Hayling test [45], and Flanker test [46].

The correlations between NSS and neuropsychological functions have significant implications. Our results indicated that the total NSS score was negatively correlated with the combined Z-score of neuropsychological tests in aMCI group. The current results were consistent with our recent findings in normal older adults that NSS had moderate associations with neurocognition function [25]. The present study provided further evidence that the two measures were more or less statistically equivalent to capture the similar brain functions. These results support in part the assumption that motor coordination might be an indicator of the prefrontal lobe function [7,8]. However, the correlation analysis also indicated that sensory integration and disinhibition did not have significant correlations with the neuropsychological tests. The reason might be as mentioned previously, sensory integration and disinhibition subscales were considered to reflect parietal and frontal lobe functions respectively [22,23], and aMCI patients showed relatively less impairment in these two subscales. Whereas, there were no sensitive neuropsychological tasks to reflect frontal lobe function and no tasks specialized to measure the parietal lobe function in current study. To better understand whether there are same neural substrates between NSS and neuropsychological functions, future studies with larger samples and more comprehensive neuropsychological tasks are needed.

The present study has several limitations. First, the sample size of the study is relatively small. More studies with larger sample sizes are needed in order to confirm the neurological dysfunctions in persons with aMCI, which could help to further clarify the early neurological abnormalities of aMCI. Second, some of the aMCI participants were from a memory clinic, and these participants were not scored by blinded raters, which might bring some bias during assessment. Future studies should overcome the difficulties and assess the NSS with blind raters. Third, aMCI participants were recruited from both community and the memory disorder clinic, while the health elderly were only selected from the community residents. This may lead to differential selection bias. Fourth, due to the mean age of participants in both groups were older than 70 years, 40 out of the 57 participants suffered from one or more chronic diseases such as hypertension, diabetes mellitus, coronary heart disease and other diseases. Also, unfortunately quite a number of the elderly could not recall the exact type and dose of medicines they have been taking during the assessment and we did not further follow up with these; therefore, we are uncertain whether their medication will influence their neuropsychological or NSS performances. Moreover, the present study adopted the traditional diagnosis criteria of aMCI proposed by Petersen and colleagues [2,26], which was called core clinical criteria by the National Institute on Aging and Alzheimer’s Association workgroup [47]. The workgroup also recommended new diagnosis criteria: research criteria, which incorporated the biomarker based on imaging or cerebrospinal fluid measures into the core clinical criteria [47]. Studies adopting more stringent research criteria for screening aMCI are needed to confirm the current findings.

Our current findings have shown that aMCI patients demonstrated significantly higher prevalence of NSS than healthy older adults. The total scores of NSS were significantly correlated with the combined Z-score of neuropsychological tests in aMCI group. NSS has been found to be indicative of the cognitive decline and brain dysfunction [4,23]. The observed impairment of NSS in aMCI contributes further evidence to the literature on neurological deficits in pathological aging diseases. Given the assessment is simple, non-invasive and time-saving, neurological soft sign test may be used as an assistant tool for the bedside clinical examination of mild cognitive impairment.

## Abbreviations

AD: Alzheimer’s disease; aMCI: Amnestic mild cognitive impairment; CNI: Cambridge Neurological Inventory; MCI: Mild cognitive impairment; MMSE: Mini Mental State Examination; NSS: Neurological soft signs; TMT: Trail-making test; WMS-RC: Wechsler Memory Scale-Revised Chinese version; WAIS-C: Wechsler Adult Intelligence Scale-Chinese version.

## Competing interests

The authors declare that they have no competing interest.

## Authors’ contributions

HJL designed the study, analyzed the data, and wrote up the first draft of the paper. JL conceived the idea and participated in the design of the study and the writing up of the paper. PYW collected the data and assisted data analysis. RCKC and YJ contributed to the writing up of the manuscript, and HLW helped aMCI participants’ recruitment and diagnosis. All authors read and approved the final manuscript.
